# From ECM Aging to Mechanobiological Restoration: Injectable Fillers and Dermal Fibroblast Mechanotransduction—A Narrative Review

**DOI:** 10.3390/gels12070609

**Published:** 2026-07-08

**Authors:** Francesco Marchetti, Mahmoud M. Fahmy Habib, Matteo Basso

**Affiliations:** 1 Casa Di Cura Villa Mafalda, 00199 Rome, Italy; 2 Department of Biomedical Engineering, Helwan University, Cairo 11795, Egypt; m.fahmy@kmedixgroup.com; 3 Mediface International School of Aesthetic Medicine, 00185 Rome, Italy; mat.bas72@gmail.com; 4 Aesthetic Medicine, Unicamillus International Medical University, 00131 Rome, Italy

**Keywords:** mechanotransduction, extracellular matrix, skin aging, YAP/TAZ, dermal fibroblast, filler rheology, hyaluronic acid, calcium hydroxylapatite, neocollagenesis, dermal remodeling

## Abstract

Skin aging reflects the accumulation of molecular damage and a progressive disruption of dermal mechanical homeostasis. Fragmentation and disorganization of the dermal extracellular matrix (ECM) impair force transmission to resident fibroblasts. Reduced cell spreading and mechanical force generation are associated with increased matrix metalloproteinase expression and reduced collagen synthesis, partly through c-Jun/AP-1 activation and attenuation of TGF-β/TβRII signaling. Reduced YAP/TAZ mechanosignaling has also been linked to cGAS-STING-dependent senescence in experimental models. However, its causal role in aging human dermis remains unresolved. This narrative review considers dermal mechanobiology as an integrative framework alongside ultraviolet exposure, oxidative stress, glycation, and cellular senescence. It examines how injectable fillers may influence the dermal mechanical microenvironment. A filler’s material properties may constitute a mechanical exposure, although bulk rheological measurements do not define force transmission at the cellular scale. Human in vivo studies of cross-linked hyaluronic acid provide the most direct evidence that enhanced structural support is associated with fibroblast spreading, activation of TGF-β-related signaling, and increased type I collagen deposition. Evidence for calcium hydroxylapatite and other biostimulatory fillers is complementary but more heterogeneous. A composite of PEGDE-cross-linked hyaluronic acid and calcium hydroxylapatite microspheres is considered as an explicitly preliminary example. This review defines the current evidence, its key limitations, and the mechanistically informed studies needed to determine whether, and which, fillers can meaningfully modify dermal mechanobiology.

## 1. Introduction

The extracellular matrix (ECM) is not only a structural scaffold. It is a dynamic platform that conveys biochemical and mechanical information to resident cells and thereby influences proliferation, migration, differentiation, and survival [1,2]. Cells sense mechanical cues through integrins and focal adhesion complexes that connect the ECM to the actin cytoskeleton. Rho GTPase-mediated contractility transmits force to the nucleus, where mechanosensitive transcriptional regulators, including yes-associated protein (YAP) and transcriptional coactivator with PDZ-binding motif (TAZ), modify gene expression in response to matrix properties [3,4,5,6,7].

In the dermis, loss of ECM structural support reduces fibroblast spreading and mechanical force generation. These changes are associated with c-Jun/AP-1 activator protein 1 activation, increased matrix metalloproteinase-1 expression, reduced TβRII expression, and lower transforming growth factor-β (TGF-β)-regulated ECM production [8,9]. Matrix stiffness alone does not determine fibroblast behavior. Local matrix architecture, ligand availability, and force transmission are also important [10]. Genetic studies in experimental models further indicate that reduced YAP/TAZ mechanosignaling can contribute to stromal tissue aging through cyclic GMP-AMP synthase and stimulator of interferon genes (cGAS-STING) activation [11]. Whether this mechanism has a causal role in aging human dermis remains unknown.

Cutaneous aging is multifactorial. Ultraviolet exposure, oxidative stress, glycation, and age-related vascular alterations contribute to dermal deterioration and have been extensively characterized within established biochemical frameworks [12,13,14,15,16,17,18]. The present review does not seek to replace these mechanisms. Instead, it examines a complementary process: progressive loss of dermal ECM integrity and its consequences for fibroblast mechanotransduction. Several established biochemical drivers, particularly UV-induced oxidative stress and matrix metalloproteinase (MMP) activity, converge on collagen fragmentation and disrupted force transmission [13,14,15]. The mechanical microenvironment may therefore serve as an integrative lens rather than as a competing explanation.

This narrative review has four aims. First, it summarizes how age-related ECM deterioration disrupts fibroblast mechanotransduction. Second, it outlines a potential self-reinforcing cascade linking mechanical decline, cellular senescence, and inflammaging, while distinguishing evidence from human dermis from findings derived from animal, ex vivo, and in vitro models. Third, it considers how the material and rheological properties of injectable fillers may act as mechanobiological exposures. Fourth, it applies this framework to a poly(ethylene glycol) diglycidyl ether (PEGDE)-cross-linked hyaluronic acid (HA) and calcium hydroxylapatite (CaHA) composite as an explicitly preliminary worked example. The review also specifies the in vivo measurements required to test, and potentially challenge, its central predictions in human dermis.

## 2. Scope and Literature Approach

This work is a narrative review and does not follow a systematic PRISMA-based protocol. Its purpose is to synthesize and interpret heterogeneous evidence rather than to provide an exhaustive or quantitatively pooled evaluation.

Relevant literature was identified through PubMed, Scopus, and Google Scholar searches conducted from April through June 2026. Search concepts included “mechanotransduction,” “dermal extracellular matrix,” “skin aging,” “YAP/TAZ,” “cGAS-STING,” “fibroblast,” “filler rheology,” “tissue integration,” “cross-linked hyaluronic acid,” “BDDE,” “PEGDE,” “crosslinker biocompatibility,” “calcium hydroxylapatite,” “neocollagenesis,” and “dermal filler.” Reference lists of eligible articles were also screened for additional sources. Priority was given to in vivo human studies, mechanistic primary research, comparative material studies, and recent authoritative reviews. Preclinical, ex vivo, and in vitro studies were included when human evidence was unavailable and are identified as such throughout the review.

Two features of the evidence base require explicit acknowledgment. First, the worked example concerns a specific material combination, and some clinical or translational sources relevant to this example [19,20,21,22,23,24] have overlapping authorship or industry affiliations with the authors of this review. The studies are interpreted alongside independent evidence and in the context of their design limitations. Second, no formal risk-of-bias assessment or sensitivity analysis excluding affiliated sources was performed. The narrative format cannot therefore exclude citation bias. The proposed framework should be interpreted as a hypothesis-generating synthesis rather than as graded, guideline-level evidence.

## 3. Multifactorial Drivers of Dermal Aging: Oxidative, Photochemical, and Glycative Contributions

The mechanobiological perspective should be considered within the broader biology of skin aging. The mechanical microenvironment is shaped by classical degradative pathways and is not independent of them. Cutaneous aging is commonly divided into intrinsic, chronological aging and extrinsic aging, with solar UV radiation as a major contributor to photoaging. These processes have distinct upstream drivers but converge on overlapping molecular and structural changes [12,13,14,25,26]. Oxidative stress, UV-induced photodamage, and glycation are major contributors to the ECM alterations that can impair dermal force transmission.

Oxidative stress is an important contributor to intrinsic aging. Reactive oxygen species (ROS) are generated during mitochondrial respiration and by external exposures, including UV radiation and air pollution. When oxidant production exceeds antioxidant defenses, damage to DNA, lipids, and proteins can accumulate. Mitochondrial dysfunction and mitochondrial DNA damage are implicated in this process, although their precise causal contribution to skin aging in humans remains incompletely defined [27]. In the dermis, ROS-associated signaling can increase matrix metalloproteinase activity and reduce collagen synthesis, thereby linking redox imbalance to ECM degradation [13,15,27,28,29]. UV exposure amplifies these processes by generating ROS, inducing inflammatory mediators, and activating AP-1-dependent matrix metalloproteinase expression [14,15]. Oxidative and photochemical injury can therefore promote collagen fragmentation and impair the structural context in which fibroblasts generate force.

Glycation adds a distinct mechanical component. The nonenzymatic reaction of reducing sugars with long-lived dermal proteins produces advanced glycation end-products (AGEs). These products accumulate in collagen and elastin and can form abnormal intermolecular cross-links [16]. A systematic review reported that AGEs are associated with altered collagen organization, reduced elasticity, and impaired remodeling. However, much of this evidence derives from diabetic, wound-healing, and scar-related contexts and should not be interpreted as direct proof for normal chronological aging alone [17]. AGE-modified collagen may become stiffer and less flexible at the molecular or fibrillar scale. It may also become more resistant to enzymatic degradation, which can impair matrix turnover [16].

Mechanical behavior at the tissue scale is more complex. It depends on collagen abundance, fibril organization, fragmentation, cross-linking, and network continuity. Consequently, measurements of molecular or fibrillar stiffness cannot be assumed to predict tissue-scale mechanics or cell-scale force transmission [10,16,30,31]. Current evidence does not support describing aged dermis as uniformly softer or uniformly stiffer. Instead, aging produces heterogeneous and scale-dependent mechanical changes.

The implication for this review is twofold. First, mechanotransduction should be viewed as one component of a multifactorial network that operates downstream of, and interacts with, oxidative, photochemical, and glycative injury. Second, ECM-directed interventions may influence several of these pathways indirectly, but they should not be assumed to reverse established nonenzymatic AGE cross-links.

## 4. Structural and Mechanical Deterioration of the Aging Dermis

Histological, morphometric, and biomechanical studies show that many clinically relevant manifestations of skin aging arise from structural changes in the dermis and its extracellular matrix [32,33]. Collagen and elastic fiber networks, together with glycosaminoglycans (GAGs) and proteoglycans (PGs), undergo age-related quantitative and structural alterations. These changes are associated with reduced skin resilience and elasticity, region-dependent changes in dermal thickness, and the progressive appearance of wrinkles [13,14,30,34].

Both the papillary and reticular dermis are affected, although the nature and magnitude of these changes vary according to anatomical site, degree of photoexposure, sex, and age [30,35,36]. In the papillary dermis, collagen density decreases and collagen fibrils become increasingly disorganized [31,35]. Age-associated reductions in hyaluronic acid, decorin, and several other GAG- and PG-related markers have also been reported. Perlecan reduction was observed in female skin in one in vivo study and should therefore not be generalized without qualification [37]. Progressive atrophy of oxytalan fibers in the superficial dermis has also been described [36].

The reticular dermis likewise undergoes age-related remodeling. Studies report changes in collagen bundle architecture, reduced collagen abundance in older age groups, and alterations in elastic fiber organization [30,35,36,38]. These changes may compromise the capacity of the dermal matrix to provide structural support for resident fibroblasts. However, direct measurements of force transmission at the fibroblast level in aged human dermis remain limited.

As outlined in Section 3, these structural alterations arise from convergent oxidative, photochemical, glycative, and cell-intrinsic processes rather than from mechanical decline alone [14,15,16,27]. With aging, hyaluronic acid shows altered tissue distribution and reduced extractability, while several regulatory PGs are reduced [37,39,40]. Type I collagen fragmentation accumulates in aged dermis [41,42], and age-related changes in the content and distribution of types I and III collagen have also been reported [43].

These changes are accompanied by measurable, but scale-dependent, alterations in dermal mechanics. In vivo measurements indicate reduced skin resilience and elasticity with aging [30]. In contrast, nanoscale analyses of aged dermal collagen have shown increased roughness, stiffness, and hardness of collagen fibrils and fiber bundles [31,44]. Tissue-level biomechanical behavior and fibril-level mechanical properties should therefore not be treated as interchangeable. This scale dependence also sets the dermis apart from the general model of tissue aging, in which ECM stiffening from collagen cross-linking and elastin loss is treated as a unifying hallmark [7]; in skin, tissue-scale softening from matrix fragmentation can coexist with fibril-scale stiffening. The net mechanical signal experienced by fibroblasts depends on matrix architecture, continuity, local composition, and cellular attachment, in addition to material stiffness.

The mechanobiological consequence is likely to be impaired fibroblast spreading and reduced mechanical force generation. In human dermal fibroblasts, reduced cell spreading and mechanical force are associated with increased MMP-1 expression, collagen fibril fragmentation, and attenuation of TGF-β receptor signaling [8,9,32]. These changes provide a plausible basis for the secondary dysregulation of mechanotransductive signaling discussed in Section 6. The principal structural and biomechanical alterations are summarized in Table 1.

## 5. Cellular Senescence, SASP, and Inflammaging

A further component of age-related dermal dysfunction involves changes in fibroblast state. Cellular senescence is a stable cell-cycle arrest that can be induced by persistent DNA damage, telomere attrition, oxidative stress, mitochondrial dysfunction, and oncogenic signaling [45,46]. Senescent cells accumulate in aging human skin, and their abundance has been reported to increase with donor age. Photoexposure can also promote senescence-associated changes. However, the magnitude of these effects varies according to anatomical site, cell type, and the biomarkers used to identify senescence [45,46].

Senescence can initially be beneficial because it limits the proliferation of damaged cells and can contribute to tissue repair. Persistent senescent cells may, however, disrupt tissue homeostasis through the senescence-associated secretory phenotype (SASP). The SASP is heterogeneous and context-dependent. It may include pro-inflammatory cytokines, chemokines, growth factors, and matrix-remodeling enzymes, including matrix metalloproteinases [45,46]. These mediators can promote chronic inflammation, ECM degradation, and paracrine senescence in neighboring cells. Senescent dermal fibroblasts may therefore contribute to low-grade chronic inflammation, often termed inflammaging, although fibroblasts represent only one component of the cellular network that shapes the aged dermal microenvironment.

The relationship between senescence-associated inflammation and innate DNA sensing has received increasing attention. cGAS detects cytosolic DNA arising from genomic instability, mitochondrial stress, or defects in nuclear-envelope integrity. Downstream STING activation can induce IRF3-dependent type I interferon signaling and NF-κB-dependent inflammatory signaling. In senescent cells, these pathways may contribute to SASP-associated inflammatory responses [7,47,48].

Gulen et al. showed that cGAS-STING signaling contributes to age-related inflammation and functional decline in mice. STING blockade suppressed inflammatory phenotypes in senescent human cells and tissues and improved tissue function in mouse models [48]. These findings strengthen the broader case for cGAS-STING as a contributor to mammalian aging. They do not, however, establish a skin-specific mechanism. The proposed link between impaired mechanotransduction and cGAS-STING activation in stromal cells derives principally from genetic experimental models of YAP/TAZ loss [11]. In that work, YAP/TAZ activity preserved nuclear-envelope integrity and limited cGAS-STING signaling. Direct demonstration that chronologically aged or photoaged human dermal fibroblasts exhibit the proposed nuclear-envelope leakage, cGAS-STING activation, and SASP that an injectable filler would reverse is, to our knowledge, still lacking.

This distinction has therapeutic consequences. Restoring the external scaffold is unlikely to reverse genuine senescence, but it can act on the larger pool of quiescent, viable fibroblasts. The mechanobiological and senescence models are thus complementary rather than competing: aged dermis contains both reversibly quiescent and irreversibly senescent cells, and any therapeutic claim must respect that heterogeneity.

## 6. The Mechanotransduction-Driven Degenerative Cascade

Dermal fibroblasts are mechanosensitive cells whose morphology and transcriptional activity depend on continuous interactions with a collagen-rich ECM [38,49]. In youthful skin, fibroblasts attach to organized collagen fibrils, generate traction forces, and maintain cytoskeletal tension that supports ECM homeostasis [3,4,5,6]. Age-associated collagen fragmentation disrupts these interactions and reduces fibroblast spreading and mechanical force generation [8,32].

Qin et al. showed that reduced fibroblast spreading and mechanical force activate c-Jun/AP-1 signaling and increase MMP-1 expression, thereby promoting collagen fibril fragmentation in three-dimensional collagen lattices [8]. Fisher et al. further demonstrated that reduced fibroblast size and mechanical force downregulate TGF-β type II receptor expression, impair TGF-β/Smad signaling, and reduce TGF-β-regulated ECM production [9]. Restoration of fibroblast size and mechanical force reversed these changes in their experimental model [9]. Together, these findings support a feed-forward model in which collagen fragmentation reduces fibroblast spreading and force generation, promotes MMP expression, and suppresses ECM synthesis, thereby favoring further matrix deterioration [8,9,32,38,49]. This model is supported by studies of human dermal fibroblasts, three-dimensional collagen lattices, and aged human skin. However, the relative contribution of each component within intact aging human dermis has not been quantitatively defined.

Two qualifications are important. First, age-related mechanical dysfunction is not solely matrix-derived. In a model of replicative senescence in primary human dermal fibroblasts, senescent cells showed decreased actomyosin-dependent cortical tension, reduced non-muscle myosin II activity, impaired traction force generation, altered viscoelastic behavior, and increased nuclear deformation [50]. These findings identify a cell-intrinsic component of mechanical dysfunction. However, they do not establish that restoring the external matrix cannot improve the function of senescent fibroblasts in vivo. Instead, intrinsic fibroblast senescence may limit the response to interventions that act primarily by improving extracellular mechanical support.

Second, YAP/TAZ signaling should not be interpreted as uniformly beneficial. YAP/TAZ activity integrates multiple mechanical and biochemical inputs, and persistent activation has been implicated in fibrotic responses across organ systems [3,51]. The therapeutic objective should therefore be restoration of physiological matrix organization and force transmission rather than indiscriminate enhancement of matrix stiffness or YAP/TAZ activity. Whether a defined therapeutic window exists for injectable fillers in human skin remains unknown.

With these qualifications, a potential link between declining mechanotransduction and chronic inflammation can be proposed. Sladitschek-Martens et al. reported that YAP/TAZ activity preserves nuclear-envelope integrity, partly through regulation of lamin B1 and ACTR2. Experimental YAP/TAZ loss compromised this protective mechanism and activated cGAS-STING signaling, with induction of senescence-associated inflammatory programs [11]. This study provides a mechanistic bridge between impaired mechanotransduction and inflammaging. However, the relevant evidence derives primarily from experimental stromal-cell and mouse models. Direct confirmation of this cascade in chronologically aged or photoaged human dermis is lacking [11,48].

**Figure 1:**
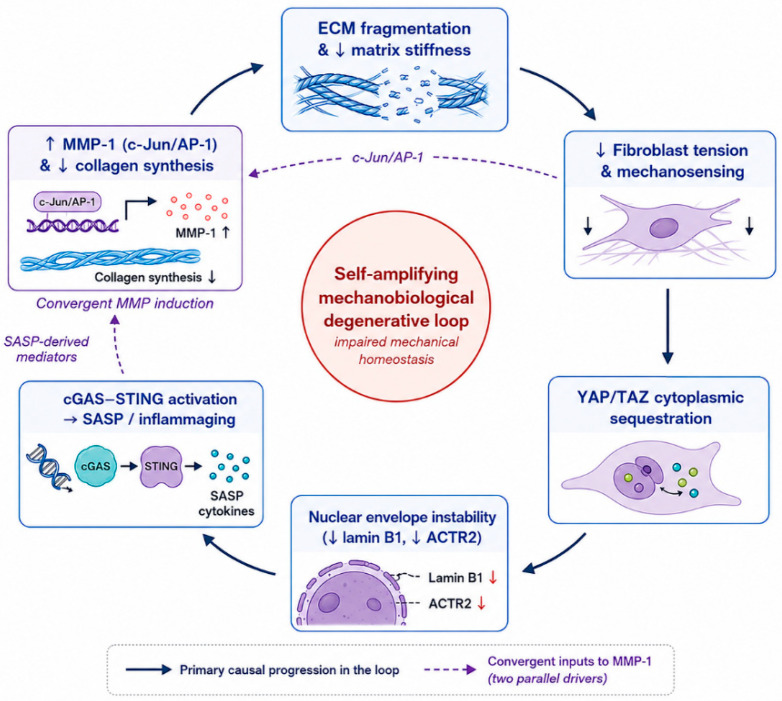
Conceptual self-amplifying mechanobiological degenerative loop in the aging dermis. ECM fragmentation reduces matrix stiffness and the tension transmitted to fibroblasts, favoring cytoplasmic sequestration of YAP/TAZ and, in animal models, nuclear-envelope destabilization with cGAS-STING activation, a senescence-associated secretory phenotype, and further MMP-driven matrix loss. Nodes downstream of YAP/TAZ are inferred largely from genetic and in vitro models and remain to be confirmed in aged human dermis.

Accordingly, the expanded model, ECM fragmentation leading to reduced fibroblast tension, reduced YAP/TAZ nuclear activity, nuclear-envelope dysfunction, cGAS-STING activation, SASP-associated inflammation, and further matrix degradation, should be presented as a hypothesis assembled across model systems. Its individual components are supported to differing degrees, but the complete sequence has not been demonstrated in aging human skin (Figure 1).

## 7. Reading Fillers Through Rheology and Complementary Material Testing: Material Properties as Mechanobiological Exposure

Injectable hyaluronic acid fillers are viscoelastic biomaterials. Their behavior should not be interpreted through a single rheological parameter. Storage modulus (G′), loss modulus (G″), loss tangent (tan δ), and viscosity describe distinct aspects of material response under defined testing conditions. Nonlinear deformation, recovery after high strain, tensile behavior, cohesivity, and susceptibility to enzymatic degradation provide complementary information [52,53,54,55].

At the material level, rheological measurements can support a plausible hypothesis that a gel will resist deformation, maintain local volume, or recover after mechanical perturbation. However, they do not directly define the mechanical environment experienced by fibroblasts in vivo. They cannot establish the local strain field, cellular traction forces, focal-adhesion tension, nuclear YAP/TAZ localization, or downstream transcriptional responses within aged human dermis. These outcomes also depend on injection depth, tissue confinement, local ECM architecture, filler volume, degradation, inflammation, and cell–matrix interactions.

PEGDE-cross-linked HA fillers merit particular consideration within this framework. PEGDE is a cross-linking chemistry distinct from 1,4-butanediol diglycidyl ether (BDDE) and may generate HA networks with a characteristic balance of structural integrity, deformability, and recovery after mechanical loading [56]. This material profile is particularly relevant for injectable products intended to perform in a dynamic, mechanically heterogeneous tissue environment. In the comparative study by Zhu et al., the evaluated PEGDE-cross-linked fillers were among the products with the highest crossover-strain values and demonstrated high recovery following the applied step-strain protocol. The modulus of cohesion derived from amplitude-sweep testing also varied substantially within this group, with one PEGDE formulation showing the highest value in the dataset [55]. Collectively, these findings support the interpretation that selected PEGDE-HA formulations can maintain network continuity during substantial deformation while recovering elastic behavior after loading.

These findings should not be interpreted as evidence that PEGDE cross-linking is universally superior, nor do they independently demonstrate direct activation of fibroblast mechanotransduction. However, rheological behavior, cohesivity, swelling characteristics, degradation profile, and in-tissue persistence together shape the physical microenvironment generated by a filler after injection. In this context, PEGDE cross-linking represents a technologically distinctive approach that may support sustained material continuity and a stable, mechanically active presence within the treated tissue.

A complementary mechanistic premise comes from tissue-engineering studies. In controlled hydrogel systems, cell spreading, matrix dimensionality, degradability, and mechanical properties can influence mechanosensitive signaling. In HA hydrogel models, stiffer two-dimensional substrates promoted cell spreading and nuclear YAP/TAZ localization, whereas three-dimensional responses depended strongly on matrix degradability and the capacity of cells to spread within the network [57]. Studies of dermal fibroblasts similarly indicate that nanoscale ECM mechanical properties can influence cell morphology and function [10]. These findings support the biological plausibility that the physical microenvironment created by an HA-based material may influence cell behavior. They do not, however, demonstrate that injectable fillers reproduce the same mechanobiological response in human dermis after clinical injection.

For candidate PEGDE-cross-linked HA formulations, including PEGDE-HA/CaHA composites, the relationship between material properties, tissue integration, fibroblast behavior, and mechanotransduction should be viewed as biologically plausible and worthy of targeted investigation. Direct demonstration would require clinically relevant in vivo or ex vivo evidence of filler distribution, local ECM remodeling, fibroblast morphology, mechanosensitive signaling, and downstream biological outcomes. Nevertheless, the available material data provide a rational basis for considering PEGDE technology as a differentiated platform for fillers designed to combine resilience, cohesivity, and adaptive mechanical performance.

## 8. Evidence Across Injectable Filler Classes Relevant to Dermal Mechanobiology

Injectable fillers may influence dermal remodeling through different, partially overlapping pathways. Cross-linked HA primarily provides local tissue support, whereas particulate fillers such as CaHA and degradable polymeric biostimulators may promote remodeling through material-specific cellular and matrix interactions. The available evidence includes human biopsy studies, clinical imaging, material characterization, and experimental models (Table 2). These data are complementary, but they do not support a single universal mechanism across all filler classes.

### 8.1. Conventional Cross-Linked Hyaluronic Acid

Conventional cross-linked hyaluronic acid (CL-HA) provides the strongest direct human evidence relevant to a structural-support-associated remodeling response. Studies in aged and photoaged skin have reported fibroblast elongation, increased type I procollagen expression, changes in TGF-β-related markers, and more organized collagen after CL-HA injection [58,59,60,61,62].

These findings are consistent with the possibility that a space-occupying HA network modifies local matrix geometry and mechanical boundary conditions, thereby supporting fibroblast spreading and collagen remodeling [58,60,62,63]. However, the available human studies do not directly measure local strain transmission, focal-adhesion loading, or YAP/TAZ signaling. CL-HA therefore provides an important clinical reference for filler-associated dermal remodeling, while the precise mechanobiological sequence remains to be defined.

### 8.2. Calcium Hydroxylapatite and Poly-L-Lactic Acid (PLLA)

Calcium hydroxylapatite is a distinct biostimulatory filler class with evidence for collagen-related remodeling, angiogenesis-related pathways, elastogenesis, and altered growth-factor signaling [64,65,66,67]. In experimental models, direct contact between CaHA microspheres and fibroblasts has also been associated with increased collagen-related activity [68]. A randomized histomorphologic study further demonstrated that CaHA and an HA-based filler may produce different tissue-remodeling profiles [69].

PLLA is a relevant comparator because it represents a degradable polymeric biostimulator whose clinical effects are generally considered gradual and associated with time-dependent tissue remodeling rather than immediate hydrogel-mediated structural support. Its inclusion illustrates that collagen remodeling may occur through biological pathways distinct from those proposed for HA-based fillers [70,71,72].

### 8.3. PEGDE-Cross-Linked Hyaluronic Acid

PEGDE-cross-linked HA represents a distinct material platform within the broader HA filler category. Available studies describe its chemical structure, network architecture, mechanical behavior, tissue-integration characteristics, and cellular-tolerance profile [23,24,56,73,74,75,76].

Comparative in vitro findings suggest that PEGDE- and BDDE-cross-linked HA systems may differ in cytotoxicity-related and oxidative-stress endpoints under defined experimental conditions [56,74,76,77,78]. In addition, a multi-parametric comparison of commercial HA fillers showed that G′ alone does not adequately describe filler behavior, highlighting the relevance of deformation and recovery characteristics when evaluating HA-based materials [55].

These observations provide a formulation-specific rationale for investigating PEGDE-HA in relation to local matrix support, tissue adaptation, and fibroblast-matrix interaction. They do not imply a universal advantage over other HA fillers. Rather, they indicate that cross-linking chemistry and formulation architecture may influence the local mechanical and biological environment and should be assessed at the product level.

### 8.4. Hybrid and Composite Systems

Hybrid fillers combine material features that are often studied separately. HA-CaHA formulations integrate a hydrogel phase with particulate components associated with collagen-related remodeling. Clinical imaging studies indicate that tissue-level responses to such formulations can be followed using ultrasound and elastography [79].

PEGDE-HA/CaHA formulations are therefore relevant as formulation-specific translational examples. Their potential importance lies in the possibility that the HA phase contributes local matrix support, while CaHA microspheres provide additional remodeling-related cues. Material and in vitro studies of such formulations provide an initial rationale for this concept [80,81].

This possibility should be evaluated directly for the specific composite rather than inferred from evidence obtained with HA or CaHA alone. The following section considers PEGDE-cross-linked HA with CaHA as a translational case example and formulates a testable hypothesis concerning fibroblast behavior, collagen organization, and dermal matrix homeostasis.

**Table 2:** Comparative summary of injectable filler classes and their relevance to dermal mechanobiology.

Filler Class	Representative Evidence	Main Observations	Scope of Inference	Ref.
Conventional cross-linked HA	Human biopsy studies in aged or photoaged skin	Fibroblast elongation, increased procollagen and TGF-β-related markers, and more organized collagen	Direct human evidence for filler-associated dermal remodeling; cell-scale force transmission and equivalence across cross-linking chemistries remain unmeasured	[58,59,60,61,62]
CaHA	Human, translational, ex vivo, and in vitro studies	Collagen-related remodeling, angiogenesis-related responses, and fibroblast activation under direct-contact conditions	Supports an independent remodeling rationale; direct microsphere-fibroblast contact in vivo has not been established	[64,65,66,68,69]
PEGDE-cross-linked HA	Comparative material and cell-compatibility studies	Formulation-dependent rheological, structural, and cellular-tolerance characteristics	Supports a material and preclinical rationale; direct human mechanotransduction evidence is not available	[23,24,56,73,74,75,76]
HA-CaHA hybrid systems	Imaging and clinical observational studies	Tissue-level outcomes can be assessed by ultrasound and elastography	Supports formulation-specific imaging and tissue-response evaluation; no common hybrid mechanism can be inferred	[79]
PEGDE-HA/CaHA composite	In vitro fibroblast, histological, safety-focused, and early clinical observations	Collagen-related fibroblast activity, tissue-integration observations, and preliminary remodeling signals	Hypothesis-generating evidence that requires controlled, mechanistically instrumented human studies	[19,20,21,23,24,80,81]

## 9. PEGDE-Cross-Linked HA with CaHA: A Translational Case Example and Testable Hypothesis

A composite formulation containing PEGDE-cross-linked HA, CaHA microspheres, glycine, and L-proline provides a useful translational case example. The formulation should not be considered representative of all PEGDE-HA or HA-CaHA products. Instead, it illustrates how the proposed mechanobiological framework can be converted into testable material, cellular, and clinical questions.

The working hypothesis may be described cautiously through a “dual-core” framework [22]. In this formulation-specific context, the term refers to the potentially complementary roles of the HA network and CaHA microspheres, rather than to a proven synergistic mechanism. The HA network may contribute to local structural support and matrix continuity, whereas CaHA microspheres may provide a localized particulate stimulus that influences fibroblast behavior and tissue remodeling. The individual roles of glycine and L-proline have not been independently tested in the available studies and cannot currently be assigned a specific mechanistic contribution. Overall, the proposed effects on fibroblast spreading, collagen organization, and matrix homeostasis remain hypotheses requiring component-resolved testing.

Material-characterization studies have described the rheological and physicochemical properties of PEGDE-cross-linked HA formulations containing CaHA [73,75,80]. Histological and ultrastructural reports have also described tissue integration of PEGDE-based HA gels and spatial continuity with adjacent dermal structures [23,24]. These observations are compatible with a potential scaffold role. However, bulk rheology and morphology cannot define the local strain field, focal-adhesion forces, or mechanosensitive signaling experienced by fibroblasts in vivo.

A direct biological observation comes from an in vitro study of human dermal fibroblasts exposed to PEGDE-cross-linked HA containing low-concentration CaHA. Soluble collagen production increased by 37.62% and 97.39% at product concentrations of 1.25 mg/mL and 2.5 mg/mL, respectively [81]. This finding supports collagen-related activity under controlled conditions. However, the study did not include matched HA-only, CaHA-only, glycine/L-proline-only, or alternative-crosslinker comparator arms. It therefore cannot identify the contribution of individual formulation components or establish additive or synergistic effects.

The CaHA component is supported by the direct-contact model described by Nowag et al. [68]. In vivo, however, direct fibroblast-microsphere contact may vary according to particle distribution, carrier-gel behavior, interstitial fluid, tissue architecture, intervening inflammatory cells, and the physical separation created by the surrounding hydrogel matrix. The cellular relevance of direct contact should therefore be assessed directly in treated human skin rather than inferred from in vitro exposure conditions.

A further consideration concerns the long-term fate of the particulate component. CaHA is generally regarded as biodegradable, but the rate and completeness of local clearance, as well as the relative contributions of residual material and newly organized matrix to persistent tissue effects, may vary by anatomical site and tissue environment. Imaging evidence has shown no detectable CaHA in one treated anatomical setting at 2.5 years, whereas an isolated histological case report identified CaHA microspheres with associated fibrosis in facial skin six years after implantation [82,83]. Rare foreign-body reactions have also been reported after CaHA injection [84]. The studies informing the present composite case example extend only to nine months. They cannot establish longer-term material persistence, local viscoelastic consequences, focal mineralized deposits, or the frequency of delayed nodular or granulomatous responses. Progressive dermal thickening within this interval should therefore not be equated with indefinitely sustained physiological remodeling.

Because the composite is placed subdermally, its potential mechanical effects may not be confined to the dermis. Subcutaneous white adipose tissue (sWAT) is a mechanically relevant compartment that may influence load distribution and support of the overlying dermis, yet its response to injected CaHA has not been systematically characterized [67]. If subdermal material alters local adipose volume, septal organization, or mechanical behavior, it could modify dermal tension and force transmission. Such effects could reinforce, mediate, or confound dermal-level observations and remain unmeasured. Future studies should therefore pair dermal endpoints with imaging and, where feasible, biomechanical assessment of the adjacent subcutaneous compartment.

Early clinical and histological observations are compatible with local tissue remodeling but remain preliminary. A case report described increased collagen-associated fluorescence after treatment with a PEGDE-HA/CaHA formulation [19]. A safety-focused clinical study provided local tissue-integration and immune-cell observations [20]. A later narrative review summarized findings from a small uncontrolled clinical series, including changes in dermal thickness and type I collagen-related outcomes [21]. These observations may inform study design, but they do not establish comparative effectiveness, component-specific effects, or a direct mechanotransductive mechanism.

The critical next step is controlled, component-resolved evaluation. Matched HA-only, CaHA-only, and composite arms, ideally with an alternative-crosslinker comparator, would be needed to test whether the composite produces additive or synergistic effects. Relevant outcomes should include filler distribution, dermal and subdermal tissue mechanics, collagen architecture, fibroblast morphology, nuclear YAP/TAZ localization, TβRII-related signaling, and selected inflammatory or cGAS-STING/SASP markers.

## 10. Measuring Filler Mechanobiology in Humans

The central claim of this framework concerns mechanical signaling. Its evaluation therefore requires methods that bridge material properties, tissue mechanics, cellular behavior, and molecular outcomes. Injectable fillers may be conceptualized as a potential form of mechanomedicine, insofar as they may modify aspects of the local mechanical microenvironment and thereby influence cell behavior. This concept is related to other approaches that use controlled mechanical loading or engineered material properties to modulate biological responses [7]. Within this framework, a favorable filler response would be expected to support physiological tissue mechanics and force transmission rather than simply maximize tissue stiffness. This is why the following measurement strategy prioritizes mechanistic endpoints alongside volumetric outcomes. Bulk rheology provides information about a filler under controlled laboratory conditions, but it cannot define the forces transmitted through focal adhesions to fibroblasts in treated human skin [55,80].

At the matrix and cellular scale, atomic force microscopy can characterize nanoscale mechanical properties of dermal ECM and collagen [10,31]. These measurements can be combined with histological assessment of collagen architecture, fibroblast morphology, and immunohistochemical markers of matrix synthesis. Relevant molecular endpoints include type I procollagen, HSP47, TβRII, CCN2/CTGF, nuclear versus cytoplasmic YAP localization, and selected cGAS-STING or SASP-related markers.

At the tissue scale, high-frequency ultrasound and elastographic methods can provide longitudinal information about dermal thickness and stiffness-related parameters. Skin stiffness measured in vivo has been proposed as a biomarker of skin aging [85]. However, elastographic and ultrasound endpoints are sensitive to anatomical site, probe pressure, operator technique, hydration, temperature, and analysis protocol. Studies should therefore include standardized acquisition procedures, baseline measurements, appropriate control sites or comparator groups, and repeated measurements to quantify intra- and inter-observer variability.

A mechanistically informative human study should integrate several scales of analysis. Material distribution and dermal thickness should be evaluated using imaging. Tissue mechanical outcomes should be assessed using validated elastographic or biomechanical methods. Biopsies, when ethically and clinically appropriate, should examine collagen organization, fibroblast morphology, and molecular markers of mechanosensitive signaling. The most informative designs would compare a PEGDE-HA formulation, a PEGDE-HA/CaHA composite, and a conventional CL-HA comparator, while controlling for injection plane, volume, anatomical site, and follow-up interval.

The desired endpoint is not a simple increase in tissue stiffness. A favorable response would be characterized by organized collagen remodeling, physiological tissue mechanics, and an absence of evidence for persistent inflammation or pathological fibrosis. This distinction is essential because matrix stiffness alone does not define a healthy mechanical microenvironment.

## 11. Limitations and Research Priorities

This narrative review integrates evidence from human skin, cell culture, ex vivo tissue, and animal models. The proposed framework should therefore be interpreted as a testable synthesis rather than a confirmed in vivo mechanism of dermal regeneration.

The principal gap is the absence of direct human evidence linking filler properties with fibroblast-scale mechanical signaling. Bulk rheology, imaging, and collagen-related outcomes cannot define local strain transmission, focal-adhesion forces, or YAP/TAZ activity in treated dermis. The contribution of cross-linking chemistry, particulate components, injection-related injury, and host tissue responses also remains unresolved.

Several publications relevant to the PEGDE-HA/CaHA case example involve overlapping author groups or industry affiliations with the present authors. These relationships are disclosed in the Conflicts of Interest section. No formal risk-of-bias assessment or sensitivity analysis excluding affiliated studies was performed.

Future controlled studies should combine assessment of filler distribution, dermal mechanics, collagen organization, fibroblast morphology, and mechanosensitive molecular endpoints.

## 12. Conclusions

Dermal mechanobiology provides an integrative framework for understanding how ECM fragmentation, impaired force transmission, fibroblast dysfunction, and matrix remodeling may interact during skin aging. This perspective complements established biochemical mechanisms of skin aging rather than replacing them.

Current evidence supports the potential of injectable fillers to influence dermal remodeling, but direct demonstration of filler-induced mechanotransduction in human skin remains limited. PEGDE-HA/CaHA composites should therefore be regarded as hypothesis-generating formulations whose proposed mechanobiological effects require controlled, formulation-specific validation.

## Figures and Tables

**Table 1:** Principal structural and biomechanical alterations of the aging dermis and their functional consequences.

Compartment	ECM Component	Age-Related Change	Mechanical/Functional Consequence	Ref.
Papillary dermis	Fibrillar collagen	Reduced collagen density and greater fibril disorganization	Reduced structural organization and potentially impaired local support for fibroblast attachment	[31,35]
Papillary dermis	Hyaluronic acid, decorin, other PGs	Reduced HA, decorin, and several GAG- or PG-related markers; perlecan reduction reported in female skin	Altered hydration, matrix organization, and cell–matrix interactions	[37,39,40]
Papillary dermis	Oxytalan fibers	Progressive atrophy of the superficial oxytalan network	Reduced contribution of the superficial elastic fiber system to tissue recoil	[36]
Reticular dermis	Collagen bundles	Age-related alterations in bundle morphology and collagen abundance	Altered load-bearing capacity and structural support of the dermis	[30,35,38]
Reticular dermis	Elastic fiber network	Age-related remodeling and disorganization of elastic fibers	Reduced elastic recovery and altered tissue mechanics	[30,36,38]
Whole dermis	Type I and III collagen	Type I collagen fragmentation; changes in collagen content and fiber distribution	Loss of matrix continuity and impaired mechanical support for fibroblasts	[41,42,43]

## Data Availability

No new data were created or analyzed in this study.
